# Missed Lisfranc injuries—surgical vs conservative treatment

**DOI:** 10.1007/s11845-020-02364-7

**Published:** 2020-09-14

**Authors:** Amit Singh, Naveen Lokikere, Aakash Saraogi, P. N. Unnikrishnan, James Davenport

**Affiliations:** 1 Royal Lancaster Infirmary, University Hospital Of Morecambe Bay NHS Foundation Trust, Ashton Road, Lancaster, Lancashire LA1 4RP UK; 2 Sports Orthopaedic Institute, Bangalore, India; 3 Sir H N Reliance Foundation Hospital, Mumbai, India; 4 The Robert Jones and Agnes Hunt Hospital U.K, Gobowen, England; 5 Wrightington Wigan and Leigh NHS Trust U.K, Wigan, UK

**Keywords:** Missed Lisfranc injury, Painful foot, Surgical vs conservative

## Abstract

**Introduction:**

Lisfranc injuries form a distinct group of rare but severe injury. Literature suggests a low incidence, but failure to diagnose these injuries early and its subsequent delay in management will affect the patient’s mobility and quality of life significantly. The preferred mode of management is said to be surgical. Conversely, the method of intervention for patients not suitable for surgery is less clear.

**Aim:**

This study aims to evaluate the effect of delayed diagnosis and the treatment provided on the overall functional outcome for the patients with missed Lisfranc injury.

**Methodology:**

The study was conducted at a specialist centre in the North-West of UK between January 2011 and November 2016. All patients with acute Lisfranc injuries were included in this study. Patient data was collected through electronic notes and analysed to ascertain missed diagnosis. It was also used to evaluate functional and radiological outcome.

**Results:**

In our series, 58.8% of Lisfranc injuries were missed on their initial presentation. We report better results for the surgical group when compared with the non-operated group, in spite of the delay in diagnosis.

**Conclusion:**

We believe that definitive treatment in the form of surgical fixation and anatomical reduction has more influence on the functional outcome than the timing of the surgical fixation in case of subtle Lisfranc injuries.

## Introduction

The tarsometatarsal joint of the foot is named as Lisfranc joint, and its injury is named after French surgeon Jacques Lisfranc [1]. This joint has complex anatomy, and the stability depends upon ligaments and bony architecture. The essential structure is the base of the second metatarsal, which is wedged into the mortise created by medial and lateral cuneiform. Although many dorsal and plantar ligament provides stability to the Lisfranc joint, the most important structure is the plantar interosseous ligament which extends from the lateral surface of the medial cuneiform to the base of the second metatarsal. This is also called a Lisfranc ligament. Lisfranc ligament is torn in pure ligamentous or in a fracture dislocation type of injury which leads to loss of alignment at tarsometatarsal joint [2]. The incidence of Lisfranc injuries is around 1 in 55,000 per year [3]. However, this number could be deceiving as approximately 20 to 24% of Lisfranc injuries are missed at their initial presentation [4]. Patient factors like reduced mobility due to associated medical comorbidities and delicate skin can influence the treatment approach to this injury [1]. Most injuries with apparent fracture and dislocation are treated surgically. However, subtle Lisfranc injuries which may appear stable on initial assessment can be missed on presentation and may receive conservative treatment. Unfortunately missed Lisfranc injuries end up with painful malunion or persistent dislocation which will require secondary arthrodesis. The current literature suggests that the outcome of secondary arthrodesis may not be satisfactory, and patients are left with significant morbidity [5]. This study has two objectives. One is to assess the functional outcome of patients who received surgical vs conservative treatment for subtle Lisfranc injury, and other is to evaluate if the delay in diagnosis of this injury has any significant effect on overall functional outcome.

## Materials and methods

We reviewed patients with Lisfranc injuries who were treated at our hospital from January 2011 to November 2016. The data collection was performed as per the hospital protocol. All the patients with acute Lisfranc injuries were included in our study. These patients were identified from their clinical, radiological, and operative notes. Due to the rarity of this injury, like many other hospitals, our hospital did not have a specific code for Lisfranc injury. Hence, all patients with metatarsal fracture were first identified. Following that, another set of data was obtained from the radiology department, which included radiographs, CT, and MRI scan reported with Lisfranc injuries. Information was also obtained from the theatre records for patients who had a fixation for Lisfranc fractures. Once all the data was available, it was crosschecked to identify the patients with true Lisfranc injury. We excluded all the patients with Charcot foot or missing data from our research. The conservative treatment was reserved for patients with stable injury or patients who are unfit for surgical intervention due to significant medical comorbidity, reduced mobility and poor skin condition. All other patients were managed surgically. Patients included in the study were followed up at 6 weeks, 3 months, 6 months and 12 months. At each visit, patients were assessed for foot pain, ability to weight bear and a radiological union of fracture/injury. The data was also analysed for complications such as infection, CRPS, stiffness and late deformity, i.e. planovalgus and arthritis.

## Results

**Fig. 1:**
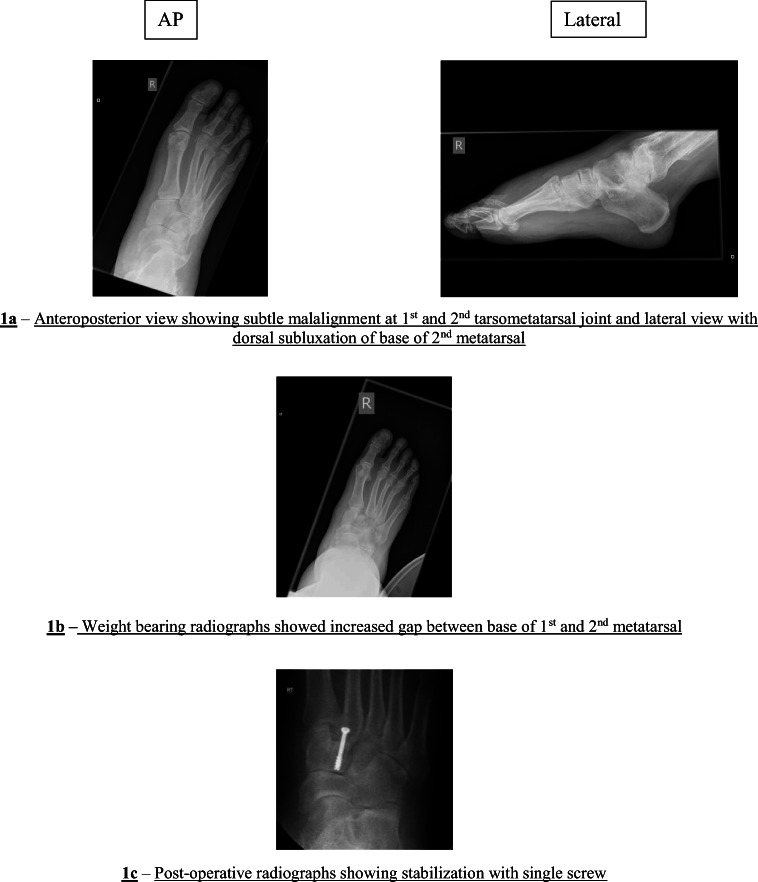
Pre- and post-operative radiographs of patient with missed Lisfranc Injury. **a** Anteroposterior view showing subtle malalignment at 1st and 2nd tarsometatarsal joint and lateral view with dorsal subluxation of base of 2nd metatarsal. **b** Weight bearing radiographs showed increased gap between base of 1st and 2nd metatarsal. **c** Post-operative radiographs showing stabilisation with single screw

We categorised our patients into two broad groups based on the treatment—operated and the conservative. Our study is a retrospective case series of 17 patients with a Lisfranc injury with a mean follow-up of 12 months (range 3 to 29 months). There were nine males and eight females with a mean age of 39.5 years (range 14 to 71). Eleven patients were managed surgically, and six were treated conservatively. Two out of 17 patients were lost to follow-up, one from each group. The surgical techniques include ORIF with plate and screws or screws alone in six (Fig. 1c), primary arthrodesis in three, open reduction and internal fixation (ORIF) with tightrope in one and closed reduction and K-wire fixation in one. Five patients had conservative management in the form of below-knee non-weight bearing plaster cast for 6 weeks followed by walking boot.

In our series, 10 (58.8%) Lisfranc injuries were missed on their initial presentation. Emergency care physicians or sports physiotherapist initially assessed all the ten patients, and later orthopaedic referral was done due to ongoing pain. These ten patients were diagnosed to have subtle ligamentous Lisfranc injuries. There was a mean delay of 6.3 weeks (range 0.6–24 weeks) in diagnosing these injuries. The Lisfranc injury was missed at initial presentation for five patients in the surgical group and five from the conservative group. The functional outcome for these patients in both the groups is defined in Table 1.

**Table 1:** Functional outcome in patients with delayed diagnosis of Lisfranc injury

Type of treatment	Total no. of patients in each group	No. of patients with missed Lisfranc injury at initial presentation	Average delay in diagnosis (weeks)	Functional outcome in patients with missed injury		Patients lost to follow-up
				Good	Poor	
Surgical	11	5	6.1	3	1	1
Conservative	6	5	6.5	1	3	1

Out of 11 patients managed surgically, 8 (80%) had an excellent clinical outcome. These patients were able to weight bear and mobilise pain-free at their last follow-up. Two patients in the surgical group continued to suffer from pain, one developed pes planus deformity, and the other showed arthritic changes with a prominent dorsal spur. None of the patients in the surgical group had an infection or any wound complications. The patient with the flatfoot deformity had a fusion of 1st and 2nd tarsometatarsal joint. Unfortunately, this was one of the patients in whom the injury was missed at initial presentation, whereas the patient with dorsal spur underwent ORIF with a screw. One patient in the surgical group was lost to follow-up.

Out of 6 patients managed conservatively, 1 (20%) patient had an excellent clinical outcome, and one patient was lost to follow-up. The rest of them continued to have pain on bearing weight at their last follow-up.

In the conservative group, initial immobilisation was done in a below-knee non-weight bearing cast for 6 weeks, and then gradual weight bearing was advised in a walker boot. One patient was lost to follow-up. Out of five, one patient was pain-free and mobilising bearing weight after treatment.

## Discussion

Midfoot injuries are challenging to treat and can be associated with devastating consequences. The results of our study highlight two main issues, the failure to recognise these injuries at initial presentation and the other one is its appropriate management.

A vast majority of midfoot injuries are commonly missed [6]. The radiograph of a foot could be challenging, and the images may require review by a specialist radiologist or an orthopaedic surgeon to avoid missing subtle injuries [7]. In one study, the majority of the patients were missed by primary physicians who may lack the experience to recognise subtle Lisfranc injuries on plain radiographs [8]. We noticed similar findings in our study as all the patients missed in our study were first seen in accident and emergency. The specialist orthopaedic team later diagnosed these patients (Fig. 1 a and b).

On reviewing the available literature, it is unclear what affects the functional outcome most, delayed diagnosis of subtle Lisfranc injury or its definitive management. Although conservative treatment has a role in subtle Lisfranc injury, the majority of the patient fail this approach and show improve functional scores following the surgical intervention [9]. In this study, six patients were managed conservatively, and by the completion of treatment, only one patient was able to mobilise pain-free.

The timing of surgery remains controversial for Lisfranc injuries which require surgical intervention. The available research shows favourable outcome following surgical intervention both before and after 6 weeks [10]. Both Hardcastle et al. [11] and Arntz C, Veith R and Hansen S. [12] in their studies had excellent functional outcome for patients who were operated within 6 weeks. Poor results after 6 weeks of injury may be due to factors such as the need for extensive soft tissue dissection, articular destruction due to malalignment and poor stabilisation because of rounding off the ligament edges [13]. However, Kuo et al. [14] had excellent functional outcome for patients in whom joint was anatomically reduced and stabilised with internal fixation. This finding was irrespective of the timing of the surgery, i.e. less than or more than 6 weeks. In this study, no significant relationship was noticed between late diagnosis and poor outcome. We also noticed that 80% of the patients in the conservative group had persistent pain at their last follow-up in comparison with a surgical group where 80% of the patients were functionally better.

Our study does have limitations. It is a retrospective study with a small cohort of patients. We may also require a longer follow-up to see if the symptoms of patients in the conservative group improved with time or worsened in the surgical group.

## Conclusion

We believe patients with subtle Lisfranc injury require detail assessment clinically and radiographically. Hence a specialist help should be sorted earlier in the direction of treatment to avoid unnecessary delay. Conservative treatment has some scope in the management of subtle Lisfranc injuries. Still, the surgical intervention has shown significantly better functional outcome and should be considered first line unless contraindicated. The results are better even for the patients with missed Lisfranc injuries.
